# Future challenges for structural integrity of high-Iintegrity components

**DOI:** 10.1098/rsta.2024.0181

**Published:** 2025-07-03

**Authors:** John Kenneth Sharples, Robert Ainsworth

**Affiliations:** ^1^ UK Forum for Engineering Structural Integrity (FESI), Preston, UK; ^2^ MACE, The University of Manchester, Manchester, England, UK

**Keywords:** structural integrity, structural stress, fusion energy

This theme issue is dedicated to the memory of Professor Peter Flewitt, who sadly passed away on 21 September 2024.

Peter worked for the Central Electricity Generating Board and its successor companies, where he held a series of senior technical management posts in the field of materials and structural integrity to support continued safe operation of nuclear and conventional power generation plants. Later in his career, Peter joined the University of Bristol, becoming Visiting Professor at the Interface Analysis Centre in the School of Physics. His research interests included the role of residual stresses on deformation, and fracture and the role of grain-interphase boundaries on the low- and high-temperature mechanical properties of metals, alloys and graphite. Peter published over 250 papers and was co-author of three books. His awards included: DSc (1980) via Imperial College London for work on the inter-relationship between microstructure and properties of materials; Colclough Medal and Prize from The Institute of Materials (1997); Plowden Award from British Nuclear Energy Society (1993); Fellowship of the Royal Academy of Engineering (1999); Honorary Professor at Beihang University, Beijing, China and Senior Fellow in the Department of Materials at Oxford University.

During his career in the electricity supply industry, Peter was a representative at the UK Technical Advisory Group on the Structural Integrity of High Integrity Plant (TAGSI).

TAGSI provides peer review and informed comment on the scientific principles used in structural integrity assessment methods and procedures and used to underpin the interpretation of materials behaviour in response to issues raised by its sponsors. The current sponsors are: EDF Nuclear Generation Ltd, Ministry of Defence (MOD), Rolls-Royce SMR Ltd and EDF Technical Client Organisation. The Office for Nuclear Regulation and the Defence Nuclear Safety Regulator attend TAGSI as Observers. TAGSI was formed in 1988 as the successor to the UK Light Water Reactor Study Group, which, from 1973 to the late 1980s, examined the structural integrity of the Pressurized Water Reactor vessel. Over the years, the UK nuclear industry has broadened the scope of TAGSI to provide an independent view on technical issues relating to structural integrity across all operating UK nuclear plants. Examples of the output of TAGSI are contained in [1–11].

Peter was also a director of the Forum for Engineering Structural Integrity (FESI) from when the forum was formed over 20 years ago.

FESI facilitates the effective implementation of engineering structural integrity technologies and methodologies across industry sectors and promotes cross-industry knowledge transfer and the development of technologies which will support safe and cost-effective engineering design and through-life reliability. FESI fulfils this role by organiszing workshops, seminars, training courses and conferences and through its publishing arm (FESI Publishing) whereby books and monographs are available. The main FESI Conference is traditionally run every two years, which in recent years has been held jointly with the Chinese Structural Integrity Consortium (CSIC). FESI currently has 22 Corporate paying members. In recent years, FESI has appointed various Technical Theme Leaders who are tasked with managing and promoting specific technical areas mainly by organiszing workshops/seminars/meetings associated with the particular theme. The FESI Student and Early Career Council (SECC) was established aroundapproximately 4 years ago and is a group who that meets regularly of more junior structural integrity practitioners from the corporate, academic and industrial FESI organiszations, several of whom perform activities in association with the main FESI Council. The purpose of these associated activities is to form a network with the more senior FESI members. Examples of the output of FESI are contained in [1,2,12–19].

Peter was instrumental in establishing, influencing and supporting all the above initiatives.

TAGSI and FESI have collaborated on the organiszation of joint seminars, e.g [1,2], in which Peter has been heavily involved. He was also heavily involved in the organiszation of a symposium entitled ‘Future Challenges for Structural Integrity of High-Integrity Components’ which was held in Manchester in April 2024. This special issue collects together papers based on some of the presentations at that symposium.

The papers cover a range of topics in the structural integrity area.

— The opening paper by Garwood *et al*. reviews and examines development of a multi-legged argument for structural integrity proposed by TAGSI in 2001.
— Wang *et al*. review the challenges and advancements in fusion structural integrity, focusing on the tokamak concept and key in-vessel components: the breeding blanket and divertor. The paper covers fusion regulations, operational environments, load cases, multi-material joints, and the creation of fusion-specific design criteria. The paper concludes with a recommended pathway for future research, development and collaboration to advance commercial fusion energy.
— Horn *et al*. discuss a TAGSI review of fatigue crack growth calculations for transient cycles. The review covers numerical approaches to fatigue crack growth evaluation using elastic-plastic cracked-body finite-element methods; the effect of load-ratio in defining an effective stress intensity factor range; and proposals for ordering and pairing transients aligned with plant operating conditions.
— The paper by Woollin, Wintle and Stone et al. examines sizes and aspect ratios of postulated defects for the failure analysis leg of a safety case for an Incredibility of Failure component. The paper investigates the adequacy of using a high capability of detection approach to define the size of postulated defects and potentially limit the aspect ratios of defects which that are postulated in a defect tolerance assessment.
— James & Ainsworth describe the treatment of secondary stress in quasi-static fracture assessments. It is shown that the elastic follow-up factor provides a means to qualitatively describe the different behaviour of secondary stresses on fracture. New methods to be included in the R6 defect assessment procedure are presented along with remaining challenges.

## Data Availability

This article has no additional data.
